# Characterization of Human Gingival Fibroblasts on Zirconia Surfaces Containing Niobium Oxide

**DOI:** 10.3390/ma8095288

**Published:** 2015-09-10

**Authors:** Young-Dan Cho, Ji-Cheol Shin, Hyung-In Yoon, Young Ku, Hyun-Mo Ryoo, Dae-Joon Kim, Do-Gyoon Kim, Jung-Suk Han

**Affiliations:** 1 Department of Periodontology, School of Dentistry, Seoul National University, Seoul 110-749, Korea; E-Mails: cacodm@hanmail.net (Y.-D.C.); guy@snu.ac.kr (Y.K.); 2 Department of Prosthodontics, School of Dentistry, Seoul National University, Seoul 110-749, Korea; E-Mails: sjcyj@snu.ac.kr (J.-C.S.); prosyhi@naver.com (H.-I.Y.); 3 Department of Molecular Genetics, School of Dentistry and Dental Research Institute, BK21 Program, Seoul National University, Seoul 110-749, Korea; E-Mail: hmryoo@snu.ac.kr; 4 Department of Advanced Materials Engineering, Sejong University, Seoul 143-747, Korea; E-Mail: djkim@sejong.ac.kr; 5 Division of Orthodontics, College of Dentistry, Ohio State University, Columbus, OH 43210, USA; E-Mail: kim.2508@osu.edu

**Keywords:** dental implant, zirconia, niobium, human gingival fibroblasts (HGFs), mucosal sealing

## Abstract

It was indicated that tetragonal zirconia polycrystal (TZP) containing yttria (Y_2_O_3_) and niobium oxide (Nb_2_O_5_) ((Y,Nb)-TZP) could be an adequate dental material to be used at esthetically important sites. The (Y,Nb)-TZP was also proved to possess its osteogenic potential comparable with those conventional dental implant material, titanium (Ti). The objective of the current study was to characterize cellular response of human gingival fibroblasts (HGFs) to smooth and rough surfaces of the (Y,Nb)-TZP disc, which were obtained by polishing and sandblasting, respectively. Various microscopic, biochemical, and molecular techniques were used to investigate the disc surfaces and cellular responses for the experimental (Y,Nb)-TZP and the comparing Ti groups. Sandblasted rough (Y,Nb)-TZP (Zir-R) discs had the highest surface roughness. HGFs cultured on polished (Y,Nb)-TZP (Zir) showed a rounded cell morphology and light spreading at 6 h after seeding and its proliferation rate significantly increased during seven days of culture compared to other surfaces. The mRNA expressions of type I collagen, integrin α2 and β1 were significantly stimulated for the Zir group at 24 h after seeding. The current findings, combined with the previous results, indicate that (Y,Nb)-TZP provides appropriate surface condition for osseointegration at the fixture level and for peri-implant mucosal sealing at the abutment level producing a suitable candidate for dental implantation with an expected favorable clinical outcome.

## 1. Introduction

Implantology, a highly specialized field in dentistry, has rapidly developed the rehabilitation of dentition. Titanium (Ti) is a widely used dental implant material because of its excellent biocompatibility and strength [1]. However, its drawback of a visible metallic and grayish color in the esthetically important zone such as the anterior part of the oral cavity has necessitated developing new implant materials that have a similar color of tooth [2].

Zirconia has been introduced as an alternative material that satisfies the criteria of biocompatibility and esthetics, while the associated phenomenon of low strength by low temperature degradation (LTD) limited its clinical applications [3,4]. In a previous study, the LTD phenomenon in zirconia was substantially reduced by addition of niobium oxide (Nb_2_O_5_) [5,6] or tantalum oxide (Ta_2_O_5_) [7]. Our previous reports also proved that LTD reinforced tetragonal zirconia polycrystal (TZP) discs containing yttria (Y_2_O_3_) and niobium oxide (Nb_2_O_5_) ((Y,Nb)-TZP) have osteogenic potential similar to that of Ti [8], and that the new aerosol deposition technique for hydroxyapatite (HA) coating is significantly effective in enhancing the osteogenic potential [9].

Biocompatibility with soft tissue at the implant interface is also crucial for obtaining a successful implant system [10]. A sufficient gingival growth and mucosal sealing around the dental implant are essential to maintain the stable connection avoiding the risk of bacterial infections [11,12]. Periodontal soft tissue is mainly composed of periodontal ligament and gingival fibroblasts [13]. Gingival fibroblasts are involved in the maintenance and production of the gingival connective tissue [14]. Poorly formed gingival connective tissue around the implant allows ease of bacterial invasion that causes inflammation resulting in marginal bone loss [15]. Hence, the formation of a tight and firm mucosal sealing between the implant and the soft tissue interface is important for a long term clinical success of the implant system [16].

It has been reported that the property of collagen fibers around the implant neck area is similar *in vivo* regardless of the type of materials (Ti and Zir) [17]. *In vitro* observation showed the presence of integrin subunits in human gingival fibroblasts (HGFs) and the morphological alteration of HGFs due to the surface roughness of Ti [18]. Surface topography is an important factor in cell attachment, adhesion, proliferation, and differentiation [19], which also affects cell orientation and migration [20]. However, the association between functional cellular activity and the surface roughness of materials is still controversial. In particular, opposite results have been reported about whether increasing cellular proliferation is dependent on a smooth surface of biomaterials [21,22] or not [23,24]. Thus, the objective of this study was to characterize cellular response of HGFs to smooth and rough surfaces of the (Y,Nb)-TZP disc, which were obtained by polishing and sandblasting, respectively.

## 2. Results and Discussion

### 2.1. Surface Characterization of Titanium and Zirconia Discs

**Figure 1:**
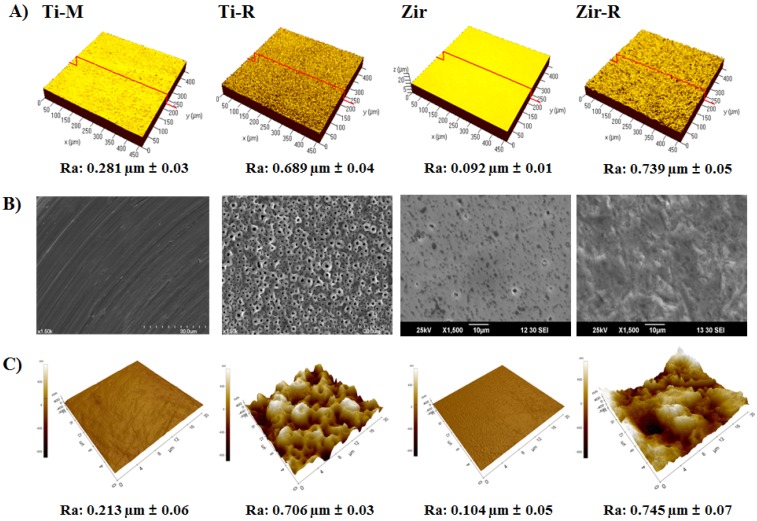
(**A**) Three-dimensional confocal laser microscopy (3D-CLM) of the examined substrate surfaces showing the roughness, *R*_a_. (**B**) Scanning electron microscopy (SEM) images at 1500× magnification, and (**C**) atomic force microscopy (AFM) images.

Proper adhesion of gingival fibroblasts to the implant surface is of importance in obtaining successful dental implantation and osseointegration [25]. Hence, surface topography is an important modulator of fibroblast adhesion [26]. As surface topography impacts cell adhesion, proliferation, and differentiation [22], the current study examined the different surface characters between Ti-machined (Ti-M), Ti-anodizing (Ti-R), (Y,Nb)-TZP (Zir) and sandblasted (Y,Nb)-TZP (Zir-R) groups The average roughness values (*R*_a_) and surface topography of the specimens were analyzed by three-dimensional confocal laser microscopy (3D-CLM) as shown in Figure 1A. The *R*_a_ values of Ti-M and Ti-R were 0.281 ± 0.03 μm and 0.689 ± 0.04 μm, respectively, and those of Zir and Zir-R were 0.092 ± 0.01 μm and 0.739 ± 0.05 μm, respectively. The *R*_a_ value of Zir was lowest due to applied fine polishing (*p* < 0.05). In order to increase the roughness to a similar level as that of titanium, sandblasting was performed with alumina particles. The conditions used for sandblasting were 50 μm alumina (Al_2_O_3_) at 2 bar pressure for 1 min.

Surface morphology was observed by using scanning electron microscopy (SEM) (Figure 1B). Machined Ti (Ti-M) showed regular scratches caused by mechanical machining operation, while the surface of anodized Ti (Ti-R) was porous and displayed crater like patterns owing to the presence of crystalline structures in the form of rutile and anatase. Polished mirror-like zirconia (Zir) showed a smooth and fine dotted pattern resulting from the process of sintering. However, after sandblasting with alumina, Zir-R exhibited uneven rough patterns. The atomic force microscopy (AFM) data showed in Figure 1C were in good agreement with the *R*_a_ values (Figure 1A). In respect of surface chemistry, blasting with Al_2_O_3_ powder may change the surface characteristics as a result of broken bonds of ZrO_2_ comprising the surface, leading to surface energy higher than as-sintered one. Thus, the adhesion is prompted to lower the energy of the system. Nevertheless, the change in composition of the surface due to the blasting is likely to be negligible since the ceramic processing to produce the specimens was including the ball milling, homogeneous heat treatment at 1250 °C and the following attrition milling, is known to provide uniform distribution of components so that the composition in bulk and surface is reasonably identical.

### 2.2. Cell Attachment and Morphology

**Figure 2:**
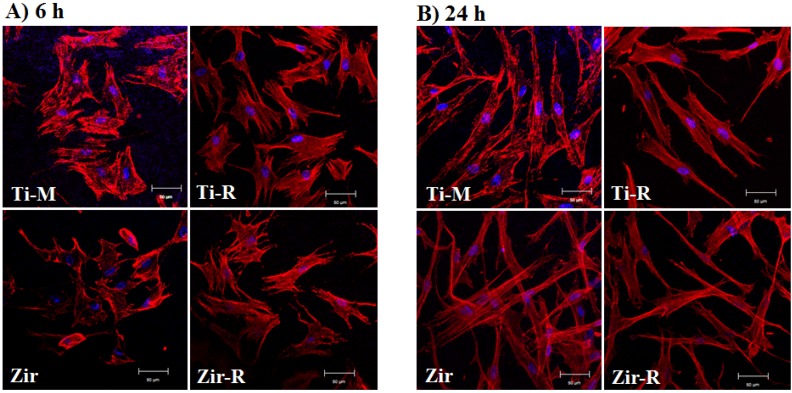
Microscopic observation after human gingival fibroblast (HGF) cells were seeded onto the discs at (**A**) 6 h and (**B**) 24 h. Original magnification is 300× and scale bar = 50 μm.

It is well established that smooth Ti surfaces favor fibroblast attachment, whereas rough Ti surfaces promote osseointegration [27,28]. In order to investigate this phenomenon between (Y,Nb)-TZP and Ti, HGFs were seeded onto the discs and harvested at 6 and 24 h, and CLM observation was followed to examine cellular attachment and morphology (Figure 2). At the early cell culture stage (6 h), cells were observed to be well attached and stretched on the Ti-M and Ti-R compared to that on Zir or Zir-R (Figure 2A). After 24 h of cell seeding, the state of cell morphology was somewhat similar between the Ti and Zir, regardless of their surface roughness (Figure 2B). Cells on the Zir were observed to be widely spread and elongated with good attachment. However, those on Zir-R were narrower and less stretched than Zir, probably indicating weaker binding with the surface.

### 2.3. Cell Proliferation

**Figure 3:**
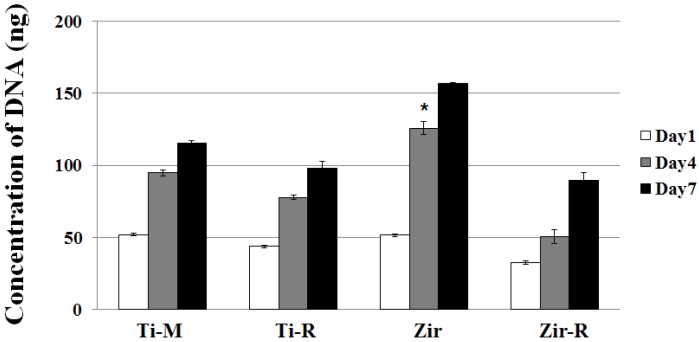
Cell proliferation assay (picogreen assay) of MC3T3-E1 cells at Day 1, 4 and 7 seeded on Ti- or Zr-discs. Data are expressed as the mean ±standard deviation (SD) of three independent experiments. Significance was tested by one-way analysis of variance (ANOVA) test (* *p* < 0.05).

Cell proliferation was evaluated by the picogreen assay. For this purpose, the HGFs were seeded onto discs and cultured for one, four, and seven days (Figure 3). HGFs were observed to proliferate well in cell cultures on all types of surfaces. The proliferation rate on Zir was significantly increased at Day 4 (*p* < 0.05) and proliferation was promoted until Day 7, indicating a positive effect of surface roughness. Generally, the smooth surfaces (*i.e.*, Ti-M or Zir) induced higher cell proliferation comparison to the rough surfaces (*i.e*., Ti-R or Zir-R). These results correlate well with those observed in case of cell attachment and morphology, which showed a more aligned and spreaded pattern in the Zir and Ti-M (Figure 2).

### 2.4. Cell Differentiation

**Figure 4:**
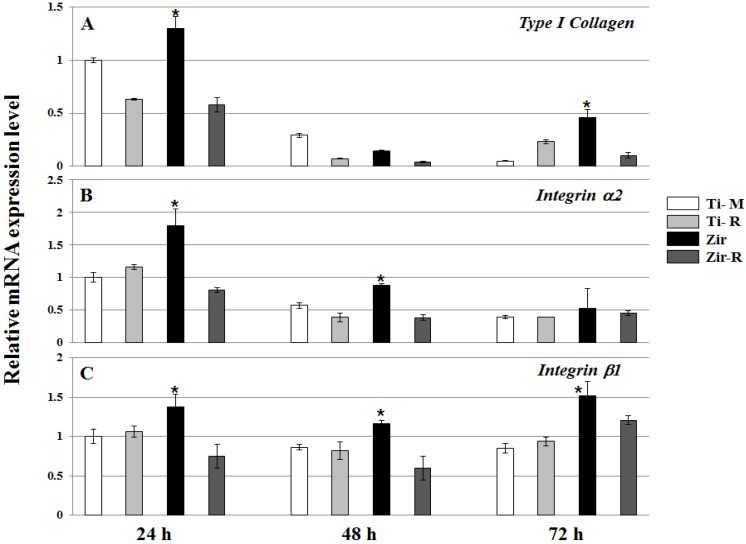
Real time polymerase chain reaction (RT-PCR) (**A**) Type I collagen, (**B**) Integrin α2, and (**C**) Integrin β1 in HGFs cultured on Ti- or Zir-discs after 24, 48 and 72 h. Data are expressed as the mean ±SD of three independent experiments. Significance was tested by one-way ANOVA test (* *p* < 0.05).

Quantitative real time polymerase chain reaction (RT-PCR) was performed to evaluate the mRNA expression level of collagen and integrin subunits. HGFs were seeded onto discs and cultured for 24, 48 and 72 h to analyze mRNA expression levels of Type I collagen, Integrin α2, Integrin β1 (Figure 4). Collagen I is mainly produced in the gingival fibroblasts, osteoblasts and periodontal ligament [29,30]. It is an important factor of gingival connective tissue and contributes to rapid periodontal tissue regeneration and maintenance of tissue architecture [31,32]. In this experiment, the mRNA levels of Type I collagen were significantly high on Zir after 24 and 72 h of cell culture (*p* < 0.05). It has been well indicated that integrins, whose α and β subunits constitute non-covalently linked αβ heterodimers, are responsible for cell adhesion [33]. Several integrin subunits including α2, α5, β1 and β3 were identified in the periodontal tissue [18,34]. Given the interplay of integrins with the extracellular matrix (ECM) and cytoskeleton [35], they regulate cellular functions, cell proliferation, adhesion, shape, and differentiation [32]. As seen in Figure 4, the mRNA levels of Integrin α2 and β1 showed almost similar patterns at each of the time points. Interestingly, Zir led to significantly high mRNA expression in Integrin α2 at 24 and 48 h, and Integrin β1 at every time point (*p* < 0.05). These results indicate that the mRNA expression of integrin subunits is correlated with cellular attachment and proliferation on the smooth Zir surface, which is partly supported by some reports [36]. In our data, Type I collagen mRNA expression was high at day 1 and then decreased (Figure 4). We guess that Type I collagen protein is well produced around one day, indicating a good attachment of gingival fibroblasts on the discs, especially Zir. In the case of Zir, cell morphology was round after 6 h of seeding; however, cytoskeleton was stretched at 24 h (Figure 2). It reflects the big increase of Type I collagen mRNA expression in the Zir. Both α1β1 and α2β1 integrins are cell surface receptors for collagens. Among them, α2β1 integrins have been shown to serve as specific receptors for type I collagen in fibroblasts, and acts as a positive regulator of type I collagen gene expression [37]. Based on these, we assume that Integrin α2 or β1 gene expression is closely related with type I collagen gene expression as our data has shown (Figure 4). In contrast with our observations, some previous studies observed no significant differences in the mRNA expression level for integrin, collagen, and fibronectin on the different disc types (*i.e.*, grooved Zir, smooth Zir, and Ti) [38].

## 3. Materials and Methods

### 3.1. Specimen Preparation

Disc shaped pure titanium specimens (25 mm diameter and 1 mm thickness) were prepared through machining (Ti-M; Ti-machined) and treated by anodizing (Ti-a; Ti-anodizing) (OnePlant System, Warrantec Co., Ltd., Seoul, Korea). Zirconia was prepared by mixing 90.6% ZrO_2_, 5.3% Y_2_O_3_, and 4.1% Nb_2_O_5_ powders for (Y,Nb)-TZP. The compositions were selected based on the absence of low temperature degradation and reasonably high fracture toughness. Disc-shaped green compacts (15 mm diameter and 1 mm thickness) were prepared by cold isostatic pressing of the powder mixtures at 200 MPa followed by sintering for 5 h at 1650 °C in air. All zirconia discs were gradually polished and finished with diamond pastes to acquire a mirror-like surface. In order to achieve roughness similar to that of Ti-R, after polishing, the (Y,Nb)-TZP was sandblasted with 50 μm alumina (Al_2_O_3_) for 1 min with 2 bar pressure.

### 3.2. Surface Roughness Assessment

The average surface roughness (*R*_a_) and surface topography were measured using a 3-D confocal laser microscope (3-D CLM; LSM700, Carl Zeiss, Oberkochen, Germany). *R*_a_ values represent the mean ±SD of three independent experiments. Surface morphology of specimens was observed using a scanning electron microscope (Ti; HITACHI S-4700, Tokyo, Japan, Zir; SNE-4500M, SEC Co., Ltd., Suwon, Korea) after sputter coating with platinum (Pt). The surface of discs was also scanned by using an atomic force microscope (AFM; XE-100, Park Systems Inc., Seoul, Korea), and the surface roughness was calculated based on the topography of the image.

### 3.3. Cell Culture

Human gingival fibroblasts (HGFs) were purchased from American Type Culture Collection (ATCC, Manassas, VA, USA), and seeded on the discs, and cultured in Dulbecco’s Modified Eagle’s Medium containing 10% fetal bovine serum and 1% penicillin/streptomycin.

### 3.4. Cell Attachment Observation

Cell attachment was observed by confocal laser microscopy (CLM, Carl Zeiss, Oberkochen, Germany). Cells were fixed on the discs using 4% formaldehyde. Hoechst 33342 (Invitrogen, Carlsbad, CA, USA) was used for detecting cell nuclei, and Alexa Fluor 568 phalloidin (Invitrogen, Carlsbad, CA, USA) was used for detecting the cytoskeleton. Fluorescence was visualized with a Carl Zeiss LSM700 microscope and analyzed with ZEN2011 software (Carl Zeiss, Oberkochen, Germany).

### 3.5. Cell Proliferation Assay

Picogreen assay was performed using the Quant-iT Picogreen assay kit (Invitrogen Ltd., Paisley, UK) at one, four and seven days after seeding the cells on the discs. Cells were washed with phosphate buffered saline (PBS) and lysed using TE buffer (10 mM Tris-HCl, 1 mM EDTA, pH 7.5). DNA concentration was determined by mixing 100 μL of Picogreen reagent and 100 μL of DNA sample. Samples were loaded in triplicate and florescence intensity was measured on a GloMax-Multi Detection System machine (Promega, Madison, WI, USA). Fluorescence intensity was converted into DNA concentration using a DNA standard curve according to manufacturer’s instructions. Values are represented mean ±SD of three independent measurements.

### 3.6. Reverse-Transcription PCR and Quantitative Real-Time PCR

Cells were harvested at 24, 48 and 72 h after seeding and RNA was isolated using QIAzol lysis reagent (QIAGEN, Valencia, CA, USA). The Primescript RT reagent kit for reverse transcription was purchased from TAKARA (Takara Bio, Shiga, Japan). Quantitative real-time PCR was performed using the primer sets for the Type I collagen, Integrin α2, and Integrin β1 according to [36]. Quantitative real-time PCR was performed using Takara SYBR premix Ex Taq (Takara Bio, Shiga, Japan) on Applied Biosystems 7500 Real Time PCR system (Foster City, CA, USA). PCR primers were synthesized by integrated DNA technology (IDT, Coralville, IA, USA). All samples were run in triplicate and the relative levels of mRNA were normalized to those of glyceraldehyde-3-phosphate dehydrogenase (GAPDH).

### 3.7. Statistical Analysis

All quantitative data are presented as the mean ±SD and each experiment was performed at least three times. Results from one representative experiment are shown. Significant differences were analyzed using ANOVA-test. A value of *p* < 0.05 was considered statistically significant.

## 4. Conclusions

Through the HGFs cellular response on the (Y,Nb)-TZP discs with different surface roughness, we proved that well-polished zirconia is superior to cell adhesion and proliferation showing increased type I collagen and Integrin α2 and β1 expression level. In comparison with Ti, zirconia shows similar cellular response in the late phase of cell culture. Based on our previous [8,9] and present data, we conclude that appropriate surface roughness of (Y,Nb)-TZP is important; rough surface for the osseointegration at the fixture level and smooth surface for the peri-implant mucosal sealing at the abutment level in the dental implant application of zirconia. As our study involves *in vitro* experiments with primary HGFs, it could have limitations for explaining general phenomenon of HGFs in view of significance.
